# Proximate causes of altitudinal differences in body size in an agamid lizard

**DOI:** 10.1002/ece3.3686

**Published:** 2017-12-03

**Authors:** Hong‐Liang Lu, Chun‐Xia Xu, Yuan‐Ting Jin, Jean‐Marc Hero, Wei‐Guo Du

**Affiliations:** ^1^ Key Laboratory of Hangzhou City for Ecosystem Protection and Restoration School of Life and Environmental Sciences Hangzhou Normal University Hangzhou China; ^2^ Key Laboratory of Animal Ecology and Conservation Biology Institute of Zoology Chinese Academy of Sciences Beijing China; ^3^ College of Life Sciences China Jiliang University Hangzhou China; ^4^ Environmental Futures Research Institute School of Environment Griffith University Gold Coast Qld Australia

**Keywords:** body size, elevational variation, growth rate, *Phrynocephalus vlangalii*, Qinghai‐Tibetan Plateau

## Abstract

Body size is directly linked to key life history traits such as growth, fecundity, and survivorship. Identifying the causes of body size variation is a critical task in ecological and evolutionary research. Body size variation along altitudinal gradients has received considerable attention; however, the underlying mechanisms are poorly understood. Here, we compared the growth rate and age structure of toad‐headed lizards (*Phrynocephalus vlangalii*) from two populations found at different elevations in the Qinghai‐Tibetan Plateau. We used mark‐recapture and skeletochronological analysis to identify the potential proximate causes of altitudinal variation in body size. Lizards from the high‐elevation site had higher growth rates and attained slightly larger adult body sizes than lizards from the low‐elevation site. However, newborns produced by high‐elevation females were smaller than those by low‐elevation females. Von Bertalanffy growth estimates predicted high‐elevation individuals would reach sexual maturity at an earlier age and have a lower mean age than low‐elevation individuals. Relatively lower mean age for the high‐elevation population was confirmed using the skeletochronological analysis. These results support the prediction that a larger adult body size of high‐elevation *P. vlangalii* results from higher growth rates, associated with higher resource availability.

## 1 INTRODUCTION

Body size is a fundamental life history trait influencing nearly every aspect of an organism's behavior and physiology and covaries with such fitness‐related traits as reproductive performance, competitive ability, and predator vulnerability (Peters, 1986; Roy, 2008; Sibly & Brown, 2007). As a central issue in ecological and evolutionary research (Angilletta, Niewiarowski, Dunham, Leache, & Porter, 2004; Peters, 1986; Stearns, 1992), numerous studies have been conducted to try to identify both proximate and ultimate causes of intra‐ and interspecies variation in body size over recent decades (Ashton & Feldman, 2003; Horváthová et al., 2013; Ryan & Smith, 2013; Valenzuela‐Sánchez, Cunningham, & Soto‐Azat, 2015).

Body size patterns for endotherms have been subject to generalizations such as Bergmann's rule, which predicts that individuals in colder regions tend to be larger than those in warmer regions (Blackburn, Gaston, & Loder, 1999). However, ectotherms in colder regions may be larger in some species, but smaller in others (e.g., Ashton & Feldman, 2003; Du, Ji, Zhang, Xu, & Shine, 2005; Forsman & Shine, 1995; Sears & Angilletta, 2004). The proximate mechanisms underlying this body size variation are complex (Angilletta, Niewiarowski et al., 2004; Sears & Angilletta, 2004). A larger body size may be a result of the following ecological scenarios. If all else being equal, (1) a larger size at birth (Kesselring, Wheatley, & Marshall, 2012; Rius, Turon, Dias, & Marshall, 2010), (2) faster growth rate (Angilletta, Steury, & Sears, 2004; Rowe, 1997), (3) longer duration of growth (delayed maturity; Angilletta, Niewiarowski et al., 2004; Horváthová et al., 2013), or (4) increased survivorship (~longevity) may lead to a larger adult body size in ectotherms with indeterminate growth (Morrison, Hero, & Browning, 2004; Norry & Loeschcke, 2002; Speakman, 2005).

Geographic variation in body size, along latitudinal gradients has been well documented in a variety of animals from insects to mammals (Du, Warner, Langkilde, Robbins, & Shine, 2010; Hodkinson, 2005; Huey, Gilchrist, Carlson, Berrigan, & Serra, 2000; Kivelä, Välimäki, Carrasco, Mäenpää, & Oksanen, 2011; Merilä, Laurila, Laugen, Räsänen, & Pahkala, 2000; Sand, Cederlund, & Danell, 1995; Sears & Angilletta, 2004; Stearns, 1992). However, variation along altitudinal gradients is not well studied, and the underlying mechanisms are poorly understood (but see Sears & Angilletta, 2003; Iraeta, Monasterio, Salvador, & Díaz, 2006; Karl, Janowitz, & Fischer, 2008). Compared with the animals living at relatively warm low‐elevation sites, high‐elevation ectotherms are expected to be larger at sexual maturity following a negative temperature–size relationship (ectotherms usually grow slower but mature at a larger size at lower rearing temperatures, e.g., Angilletta & Dunham, 2003), which is the case in some species (Angilletta, Steury et al., 2004; Morrison & Hero, 2003; Pincheira‐Donoso, Hodgson, & Tregenza, 2008; Walters & Hassall, 2006). However, body size has been found to decrease with increased elevation in other species, which has been linked to resource limitations, and thus restricted growth (Chown & Klok, 2003; Hodkinson, 2005).

The Qinghai toad‐headed lizard (*Phrynocephalus vlangalii*), which is widely distributed in the Qinghai‐Tibetan Plateau with an elevation range from 2,000 m to 4,500 m, is an excellent model species for studying altitudinal patterns of life history traits in reptiles. Altitudinal variation in body size of this species has been reported in previous studies; however, the results of these studies remain controversial. Jin, Liu, and Li (2007) found a negative relationship between body size of adult *P. vlangalii* and elevation, but a recent study showed that the females from high‐elevation sites were significantly larger than those from low‐elevation sites (Li, Zhou, & Liu, 2014). Despite the controversy in altitudinal pattern in body size, the underlying proximate causes of this altitudinal variation in body size remain elusive.

Here, we first compared the body size of *P. vlangalii* from two sites at different elevations (Maqu, 2,930 m elevation, hereafter the low‐elevation site; Maduo, 4,250 m elevation, hereafter the high‐elevation site) in the northeast part of Qinghai‐Tibetan Plateau. Then, we measured the size of newborns produced by wild‐caught females, measured growth rate and determined age structure via mark‐recapture experiments, and identified adult age by skeletochronological analysis in these two populations. We found that body size was slightly larger in the high‐elevation population than in the low‐elevation population. In order to identify the proximate causes of altitudinal variation in body size in this species, we tested the following predictions derived from the aforementioned hypotheses using data on neonate size, growth rate, estimated age at maturity, and age structure of adults, respectively. First, if neonate size determines adult body size, newborns would be larger in the high‐elevation population than the low‐elevation population. Second, if a faster growth rate leads to larger adult body size, juvenile lizards would grow more rapidly in the high‐elevation population than the low‐elevation population. Third, if lizards grow to larger body size through delayed maturity, lizards from the high‐elevation population would have a longer duration of growth than the low‐elevation population. Lastly, if increased survivorship (~longevity) leads to a larger adult body size, adult lizards would survive longer and would be older in the high‐elevation population than the low‐elevation population.

## 2 MATERIALS AND METHODS

### 2.1 Study species and areas

*Phrynocephalus vlangalii* is a small ground‐dwelling viviparous agamid lizard (up to 80 mm snout‐vent length, SVL) and typically found in open spaces among sparse vegetation in arid or semiarid regions of the Qinghai‐Tibetan plateau. Many life history traits of this species show large amounts of geographic variation. Courtship occurs in May, and parturition occurs between mid‐July and late August (Wu, Fu, Yue, & Qi, 2015). Females at low‐elevation sites give birth earlier than those at high‐elevation sites (Li et al., 2014). Offspring mass increases, but litter size and adult body size decrease with increasing elevation (Jin & Liu, 2007; Jin et al., 2007).

**Figure 1:**
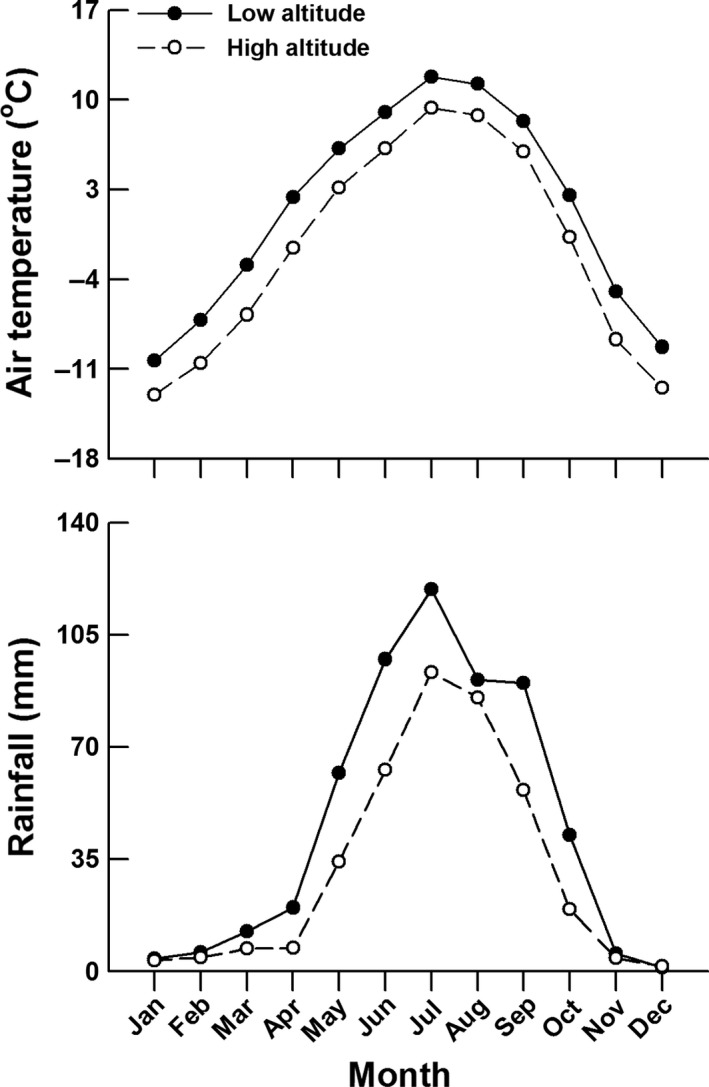
Monthly mean air temperature and rainfall for the two sites where *Phrynocephalus vlangalii* were collected (data from http://data.cma.cn/)

This study was conducted in a low‐elevation site (Gansu Province, western China, 34°00′N, 102°04′E) and a high‐elevation site (Qinghai Province, western China, 34°55′N, 98°12′E). Over a linear distance of 370 km, these two sites show distinct mean annual air temperature (low‐elevation vs. high‐elevation: 1.4°C vs. −1.7°C; paired‐sample *t* test, *t *=* *18.79, *df *= 11, *p *<* *.001, Cohen's *d *=* *0.38) and rainfall (550 mm vs. 379 mm, *t *=* *3.62, *df *= 11, *p *<* *.01, Cohen's *d *=* *0.36; Figure 1). Toad‐headed lizards are abundant in both study sites, and previous phylogenetic analysis has indicated that these two populations belong to a single lineage (Jin, Brown, & Liu, 2008).

### 2.2 Mark‐recapture experiments in the field

In mid‐June (10th–18th) and late August (22nd–30th) of 2011 and 2012, a study plot of 4,000 m^2^ in each site (80 × 50 m^2^) was visited four times. At each visit, active lizards were captured by hand whenever possible, weighed and measured for SVL, noting reproductive condition. The gender of each individual was determined by gently pressing on the tail base using thumb for the presence or absence of hemipenes. Each visit lasted approximately 1 week. A total of 1,096 lizards (146 juveniles and 366 adults from the low‐elevation, 213 juveniles and 371 adults from the high‐elevation) were captured between 2011 and 2012. Some juveniles (low‐elevation: 58 in 2011 and 75 individuals in 2012; high‐elevation: 107 in 2011 and 130 individuals in 2012) and adults (low‐elevation: 45 in 2011 and 64 individuals in 2012; high‐elevation: 34 in 2011 and 65 individuals in 2012) were marked individually by toe‐clipping upon first capture. Lizards were measured, checked for the marks, and released immediately at their site of capture. A total of 132 lizards (low‐elevation: 11 juveniles and 9 adults in 2011, 19 juveniles and 17 adults in 2012; high‐elevation: 10 juveniles and 11 adults in 2011, 41 juveniles and 14 adults in 2012) were recaptured at least once during the mark‐recapture experiment.

### 2.3 Body size of newborns

In mid‐July of 2011, 59 gravid females captured in the field (29 from the low‐elevation and 30 from the high‐elevation) were transferred to a laboratory in Hangzhou Normal University. The females were randomly housed in twelve 60 × 40 × 30 cm terraria (4–5 females per terrarium) filled with a 20 cm depth of moist sand. Terraria were housed in an AAPS (artificial atmospheric phenomena simulator) room at 20 ± 2°C on a natural light cycle. A 60‐W light bulb was suspended above one end of each terrarium (20 cm above the terrarium floor) to provide supplementary heating from 0900 to 1700 hr. Food, that is, mealworms (larvae of *Tenebrio molitor*) and house crickets (*Achetus domesticus*), and water enriched with vitamins and minerals were provided ad libitum. Parturition occurred between late July and mid‐August. All newborns were weighed and measured for SVL, and individually marked using the toe‐clipping method. Fifty‐four females (26 from the low‐elevation and 28 from the high‐elevation) gave birth to young that were all well developed, whereas the remaining females produced litters with various numbers of stillborns. The mean values for body size (SVL and mass) of newborns from a single litter were used in the following analyses to avoid pseudoreplication, and the abnormal litters were excluded from analyses. All females and juveniles were released at the site of maternal capture.

### 2.4 Skeletochronological analysis

Skeletochronology was used to assess individual age of *P. vlangalii* following Guarino, Di Già, and Sindaco (2010) and Dubey, Sinsch, Dehling, Chevalley, and Shine (2013). The third phalanxes of longest toes of the right hind limbs of some captured adult lizards (*N *=* *25 from the low‐elevation and 19 from the high‐elevation) were clipped in mid‐July of 2011 and stored in 10% neutral‐buffered formalin. Each individual digit was cleaned from surrounding tissues of the phalanx, decalcified in a 5% nitric acid solution from 24 to 48 hr, and then stained for 200 min in Harris's hematoxylin. Stained bones were dehydrated using increasing ethanol concentrated solutions. Then, phalanxes were prepared for embedding in small paraffin blocks. Phalanx diaphysis cross sections (13 μm) were obtained by means of rotary microtome. Bone sections were observed under an optic microscope (Olympus BX40, Tokyo, Japan) equipped with a Pro‐Series High Performance CCD Camera. Digital pictures were taken at 400× magnifications, and the number of lines of arrested growth (LAGs, the dense lines between bone growth zones after hematoxylin staining) was counted by two persons with previous experience of the technique. The endosteal resorption of LAGs, resulting from the replacement of periosteal bone with endosteal bone, was confirmed by determining the presence of the Kastschenko line (the division line between the endosteal and periosteal zones).

### 2.5 Data analysis

We calculated an index of body condition using the residuals from a linear regression of ln‐transformed body mass against ln‐transformed SVL, and size‐specific and mass‐specific growth rates during the mark‐recapture experiments using the formula ln(measurement_2_/measurement_1_)/(date_2_ − date_1_). The von Bertalanffy growth equation [a standard form: *L*_*t*_ = *L*_∞_ **– (*L*_∞_ **– *L*_0_)*e* ^*−Kt*^, where *L*_*t*_ is the SVL at age *t*,* L*_∞_ is the theoretical maximum length at infinite age, *L*_0_ is the initial SVL, and *K* is a growth constant that describes the rate at which *L*_∞_ is attained] is assumed to be appropriate to describe lizard growth (El Mouden, Znari, & Brown, 1999; Schoener & Schoener, 1978). We calculated von Bertalanffy growth parameters from Ford–Walford plots for each population, which were constructed by regressing final (August 2012) SVL against initial (August 2011) SVL. Data from the mark‐recapture study included individuals of 26.9–55.5 mm and 24.5–57.5 mm for the low‐elevation and high‐elevation populations, representing the upper 76% and 80% of the full‐length ranges in the two populations, respectively. The values of *L*_∞_ and *K* were calculated from the following equations: *L*_∞_ = α/(1–β) and *K *= −ln β, where α is the *y*‐intercept and β is the slope of Ford–Walford plots. Then, we used the standard and transformed [*R *= d*S*/d*t* = *K*(*L*_∞_ **– *L*_*t*_)] von Bertalanffy growth equations using the mean SVL of newborns of each population as the body size at age of 0 year (*L*_0_) to estimate SVLs and growth rates at each age, respectively. Additionally, we used the transformed equation, *t *= ln[(*L*_∞_ **– *L*_*t*_)/(*L*_∞_ **– *L*_0_)]/*–K*, to estimate the age for each individual captured in the field and at sexual maturity, using the recorded SVLs of the smallest reproductive females of each population.

Between‐sex differences in body size and growth rate of juvenile lizards were ignored because the gender of juveniles was difficult to identify using an uninjured technique. Accordingly, one‐factor analysis of variance (ANOVA) was used to determine between‐site difference in body size of field‐captured juveniles and newborns, or age estimated by skeletochronology. Two‐factor ANOVA was used to determine the differences in body size and estimated age of field‐captured adults between populations and sexes, or differences in growth rate of recaptured juveniles between populations and years. Three‐factor ANOVA was used to determine the differences in growth rate of recaptured adults between populations, sexes, and years. One‐ (or two‐) factor analysis of covariance (ANCOVA) was used to determine the differences in mass between populations (and between sexes) after removing the effect of SVL. The values of Cohen's *d* for *t* test and partial eta‐square (η_p_^2^) for ANOVA (or ANCOVA) were presented as the measures of effect size to indicate the standardized difference and the proportion of effect variance in the total variance, respectively. A value of Cohen's *d* below 0.2 or η_p_^2^ below 0.01 is considered as negligible, between 0.2 and 0.5 or between 0.01 and 0.06 as small, between 0.5 and 0.8 or between 0.06 and 0.14 as medium, and >0.8 or 0.14 as large (Cohen, 1988). Prior to parametric analyses, the normality of distributions and homogeneity of variances in the data were tested using the Kolmogorov–Smirnov test and Bartlett's test, respectively. Throughout this article, values are presented as mean ± standard error (*SE*), and the significance level is set at α = .05.

## 3 RESULTS

### 3.1 Body size of field‐captured lizards

**Figure 2:**
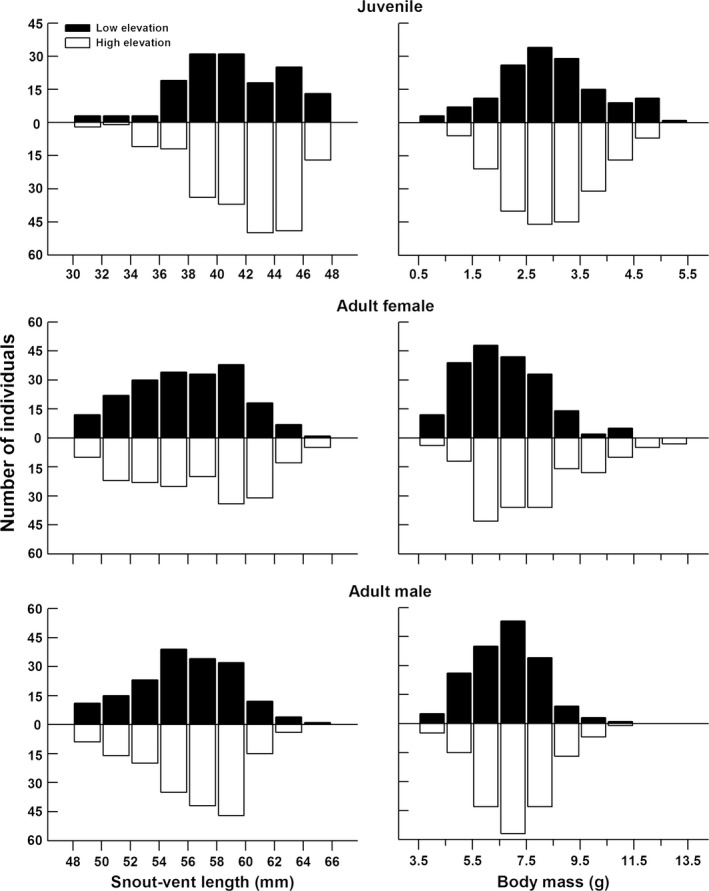
Snout‐vent length and mass distributions of *Phrynocephalus vlangalii* captured from the different elevation sites in 2011 and 2012

Juveniles from the low‐elevation site were slightly smaller but not lighter than those from the high‐elevation site (low‐elevation vs. high‐elevation, SVL: 41.1 ± 0.3* *mm vs. 41.9 ± 0.2 mm, one‐factor ANOVA with site of origin as the factor: *F*_1, 357_ = 4.66, *p *=* *.031, η_p_^2^ = 0.013; mass: 2.95 ± 0.08 g vs. 2.96 ± 0.06 g, *F*_1, 357_ = 0.01, *p *=* *.939, η_p_^2^ < 0.001; Figure 2). The mean body mass of low‐elevation juveniles was greater than that of high‐elevation ones after removing the effect of SVL (one‐factor ANCOVA with site of origin as the factor: *F*_1, 356_ = 5.10, *p *=* *.025, η_p_^2^ = 0.014). Body size (two‐factor ANOVA with site of origin and sex as the factors: *F*_1, 733_ = 1.11, *p *=* *.293, η_p_^2^ = 0.002) and mass (*F*_1, 733_ = 3.71, *p *=* *.054, η_p_^2^ = 0.005) of adult lizards did not show between‐sex differences. There was an appreciable difference in adult body size between sites. Adults from the low‐elevation site were smaller and lighter than those from the high‐elevation site (SVL, 55.8 ± 0.2 vs. 56.5 ± 0.2, *F*_1, 733_ = 6.70, *p *=* *.010, η_p_^2^ = 0.01; mass, 6.70 ± 0.07 vs. 7.39 ± 0.09, *F*_1, 733_ = 36.29, *p *<* *.001, η_p_^2^ = 0.047; Figure 2). Between‐site difference in body mass was still obvious after removing the effect of SVL (*F*_1, 732_ = 44.45, *p *<* *.001, η_p_^2^ = 0.057). Body mass of adults was affected by the interaction of site of origin × sex, with greatest mean value for high‐elevation females but smallest for low‐elevation males (*F*_1, 733_ = 10.36, *p *<* *.01, η_p_^2^ = 0.014). Adult SVL was not affected by the interaction of site of origin × sex (*F*_1, 733_ = 0.93, *p *=* *.334, η_p_^2^ = 0.001). Adults from the low‐elevation site had worse body conditions than those from the high‐elevation site (–0.031 ± 0.006 vs. 0.030 ± 0.008, *F*_1, 733_ = 38.08, *p *<* *.001, η_p_^2^ = 0.049), but juveniles did not (0.022 ± 0.021 vs. –0.015 ± 0.012, *F*_1, 357_ = 2.76, *p *=* *.098, η_p_^2^ = 0.008).

### 3.2 Growth of lizards in the field

**Figure 3:**
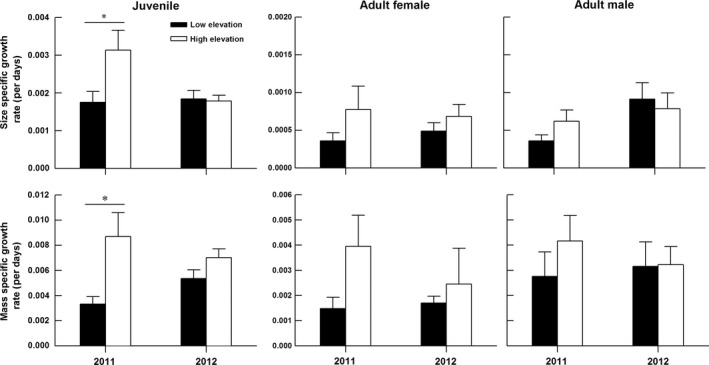
Mean values (+*SE*) of size‐ and mass‐specific growth rates of juvenile and adult *Phrynocephalus vlangalii* at different elevations in the field mark‐recapture experiments. The asterisks (*) indicate significant differences (*p* < 0.05)

Overall, juveniles at the high‐elevation site grew faster than those at the low‐elevation site (Table 1, Figure 3). Juveniles increased their SVL (but not mass) more rapidly in 2011 than in 2012 (Table 1). The year × site of origin interaction had a significant effect on size‐specific growth rate, but not on mass‐specific growth rate of juveniles (Table 1). The mean value of juvenile size‐specific growth rate in 2011 was highest at the high‐elevation site, but lowest at the low‐elevation site (Figure 3). Juvenile lizards increased their SVL (*F*_1, 124_ = 74.94, *p *<* *.001, η_p_^2^ = 0.377) and mass (*F*_1, 124_ = 21.26, *p *<* *.001, η_p_^2^ = 0.146) more rapidly than adult lizards (Figure 3). Both size‐specific and mass‐specific growth rates of recaptured adults did not differ between years, between sites, and between sexes and were not affected by the interactions of these factors (Table 1).

**Table 1:** Results of two‐factor (with year and site of origin as the factors for juveniles) or three‐factor (with year, site of origin, and sex as the factors for adults) ANOVAs on specific growth rate of *Phrynocephalus vlangalii* at different elevations in the field mark‐recapture experiments

	Juvenile		Adult	
	Size‐specific growth rate	Mass‐specific growth rate	Size‐specific growth rate	Mass‐specific growth rate
Year	*F*_1, 77_ = 4.95, *p* = .029, η_p_^2^ = 0.060	*F*_1, 77_ = 0.03, *p *=* *.858, η_p_^2^ = 0.0004	*F*_1, 38_ = 2.08, *p *=* *.157, η_p_^2^ = 0.052	*F*_1, 38_ = 0.42, *p *=* *.521, η_p_^2^ = 0.011
Site of origin	*F*_1, 77_ = 5.48, *p *=* *.022, η_p_^2^ = 0.066	*F*_1, 77_ = 11.04, *p *<* *.01, η_p_^2^ = 0.125	*F*_1, 38_ = 1.97, *p* = .169, η_p_^2^ = 0.049	*F*_1, 38_ = 2.81, *p *=* *.102, η_p_^2^ = 0.069
Sex			*F*_1, 38_ = 0.48, *p *=* *.491, η_p_^2^ = 0.013	*F*_1, 38_ = 1.75, *p *=* *.193, η_p_^2^ = 0.044
Year × site of origin	*F*_1, 77_ = 6.52, *p *=* *.013, η_p_^2^ = 0.078	*F*_1, 77_ = 3.07, *p *=* *.084, η_p_^2^ = 0.038	*F*_1, 38_ = 1.31, *p *=* *.260, η_p_^2^ = 0.033	*F*_1, 38_ = 1.20, *p *=* *.281, η_p_^2^ = 0.031
Year × sex			*F*_1, 38_ = 1.64, *p *=* *.207, η_p_^2^ = 0.041	*F*_1, 38_ = 0.07, *p *=* *.793, η_p_^2^ = 0.002
Site of origin × sex			*F*_1, 38_ = 0.81, *p *=* *.373, η_p_^2^ = 0.021	*F*_1, 38_ = 0.39, *p *=* *.535, η_p_^2^ = 0.010
Year × site of origin × sex			*F*_1, 38_ = 0.10, *p *=* *.753, η_p_^2^ = 0.003	*F*_1, 38_ = 0.02, *p *=* *.890, η_p_^2^ = 0.0005

### 3.3 Body size of reproductive females and newborns

Females in the laboratory produced 54 clutches (26 from low‐elevation site, 28 from high‐elevation site) between late July and mid‐August. The smallest reproductive females were 51.7 mm and 49.9 mm SVL for the low‐elevation and high‐elevation populations, respectively. Female SVL did not differ between the two populations (low‐elevation vs. high‐elevation, 56.6 ± 0.5 vs. 57.2 ± 0.6, one‐factor ANOVA with site of origin as the factor: *F*_1, 52_ = 0.47, *p *=* *.497, η_p_^2^ = 0.009). The mean value of neonate size was greater for the low‐elevation population (SVL: 27.4 ± 0.2 mm; mass: 0.87 ± 0.01 g) than that for the high‐elevation population (SVL: 25.1 ± 0.2 mm; mass: 0.73 ± 0.01 g; SVL, *F*_1, 52_ = 63.90, *p *<* *.001, η_p_^2^ = 0.551; mass, *F*_1, 52_ = 50.91, *p *<* *.001, η_p_^2^ = 0.495). The body condition of newborns did not differ significantly between the two populations (0.008 ± 0.014 vs. −0.007 ± 0.012, *F*_1, 52_ = 0.68, *p* = .413, η_p_^2^ = 0.013).

### 3.4 Age and growth rate estimation using the von Bertalanffy growth equation

**Figure 4:**
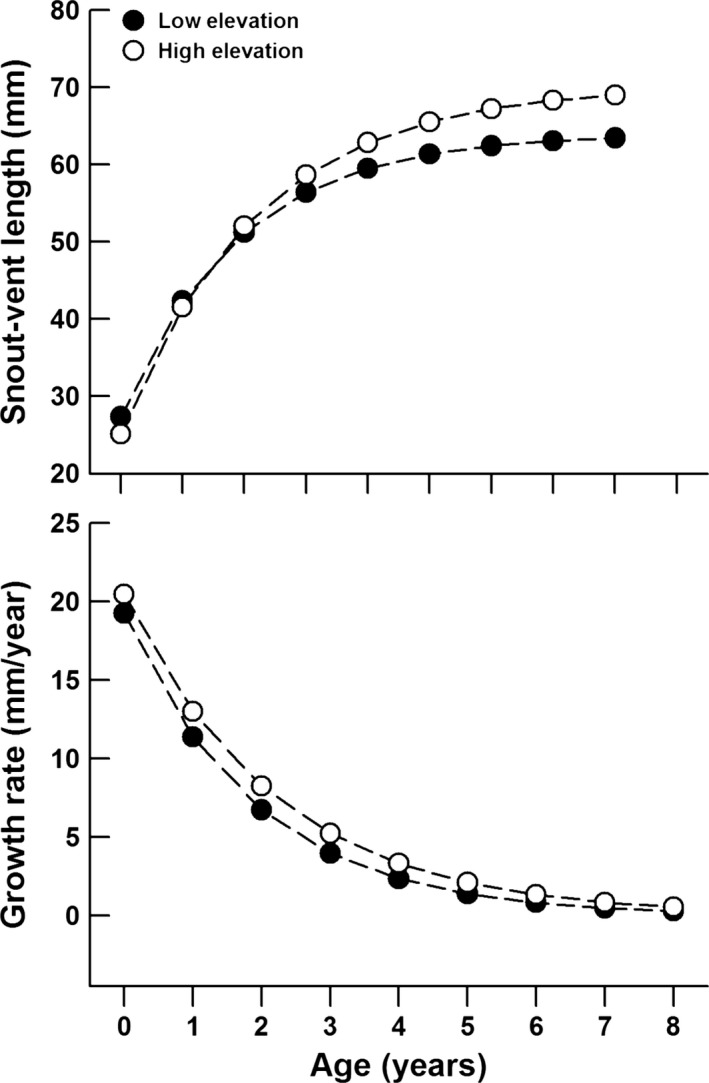
The estimated snout‐vent lengths and annual growth rates at each age using the von Bertalanffy growth equation

**Figure 5:**
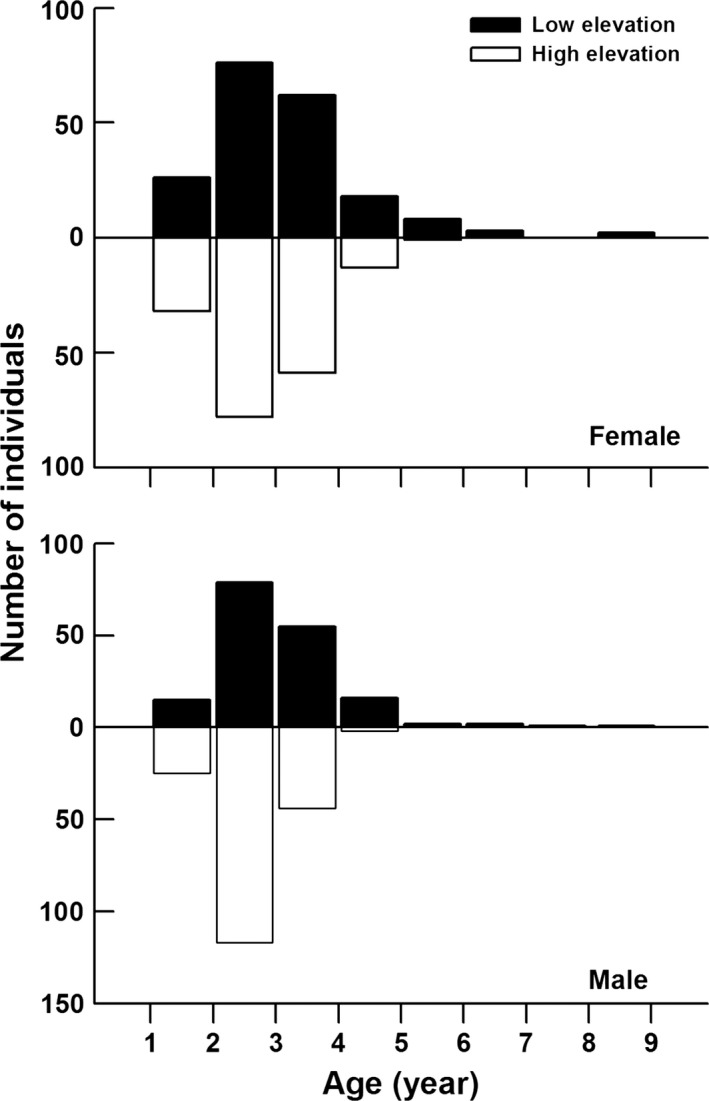
The estimated age of adult lizards captured from different elevation sites using the von Bertalanffy growth equation

The von Bertalanffy growth parameters, theoretical maximum length (*L*_∞_) and the growth constant (*K*), derived from simulated mark–recapture datasets were 64.0 and 0.53, 70.2, and 0.45 for the low‐elevation and high‐elevation populations, respectively. Estimated age at sexual maturity for the low‐elevation and high‐elevation populations was 2.1 and 1.8 years, respectively. Estimated SVLs of high‐elevation lizards were larger than those of low‐elevation ones over the age of 2 years, and estimated growth rates of high‐elevation lizards were higher than those of low‐elevation ones at each age (Figure 4). Mean estimated age of field‐captured adults was greater at the low‐elevation site (3.13 ± 0.07 years) than that at the high‐elevation site (2.72 ± 0.03 years; two‐factor ANOVA with site of origin and sex as the factors: *F*_1, 733_ = 32.18, *p *<* *.001, η_p_^2^ = 0.042), but did not differ between sexes (*F*_1, 733_ = 1.59, *p *=* *.208, η_p_^2^ = 0.002; Figure 5).

### 3.5 Age estimation by skeletochronology

Age determination by bone layers showed that the mean age for individuals from the low‐elevation site (3.68 ± 0.24 years, ranging from 2 to 6 years) was greater than that from the high‐elevation site (3.00 ± 0.22 years, ranging from 2 to 5 years; one‐factor ANOVA with site of origin as the factor: *F*_1, 42_ = 4.24, *p* = .046, η_p_^2^ = 0.092).

## 4 DISCUSSION

As reported in other lizards (Grant & Dunham, 1990; Iraeta, Salvador, & Díaz, 2013; Leache, Helmer, & Moritz, 2010; Mathies & Andrews, 1995; Rohr, 1997; Sinervo & Adolph, 1994; Sorci, Clobert, & Belichon, 1996), significant altitudinal variations in life history traits of *P. vlangalii* were showed in this study. Interestingly, low‐elevation lizards were larger at birth, but smaller in adulthood than high‐elevation ones (despite small difference in the mean values of adult SVL and mass). As a consequence of adaptation to local environments, probably, larger neonate size is advantageous for increasing survival probability in low‐elevation but slow‐growth environments (Sinervo, 1990; Warner & Andrews, 2003), while larger adult size is conducive to improving heat conservation and maintaining body temperature in colder high‐elevation environments (Olalla‐Tarraga, Rodriguez, & Hawkins, 2006; Partridge & Coyne, 1997).

In earlier studies of *P. vlangalii*, offspring size was believed to increase with increasing altitude (Jin & Liu, 2007; Li et al., 2014). This difference between the results of these studies may be due to differences in measurement methods for reproductive traits or population sampling. In the Jin and Liu (2007) study, offspring size of *P. vlangalii* was assessed using the mass of scaled embryos that removed from pregnant females, rather than the mass of newborns. Scaled embryos continued to grow before parturition, consequently, offspring size might be underestimated. In fact, mean mass of embryos from the low‐elevation population (0.89 g) was slightly larger than that from the high‐elevation population (0.85 g), if others were excluded. The Li et al. (2014) study included the high‐elevation population that studied here, but not the low‐elevation population. Therefore, these results from different studies may not be contradictory to each other and indicate that life history responses to local environments may be more complicated than expected and vary with site and year of the study (Angilletta, Steury et al., 2004; Sears & Angilletta, 2004; Stearns, 1992). That larger adult size for high‐elevation lizards than low‐elevation ones was also observed in the Li et al. (2014) study.

Newborns from high‐elevation population were smaller than those from low‐elevation population, which was inconsistent with our first prediction. If between‐site difference in adult body size of *P. vlangalii* resulted from newborn size variation, the opposite pattern should be produced. Offspring size is assumed to affect animal growth, survival, and size at sexual maturity (Marshall & Keough, 2008; Räsänen, Söderman, Laurila, & Merilä, 2008). Large offspring are favored in poor‐growing (such as low temperature, food scarcity) environments (Sinervo, 1990; Warner & Andrews, 2003). Why did not lager newborns occur in relatively colder, high‐elevation environment in *P. vlangalii*? One possibility is that environmental condition at the high‐elevation site is not as disadvantageous as expected for lizard growth. In fact, another study showed that potential prey availability at high‐elevation site was more abundant and led to higher growth rates for juveniles, than at low‐elevation site (Lu et al., in review). Accordingly, offspring size is not likely to be an important source of variation in adult body size of *P. vlangalii*.

Consistent with our second prediction, high‐elevation lizards grew more rapidly than low‐elevation ones. The growth rate of lizards may be immediately affected by food availability (Iraeta et al., 2013). The high‐elevation site could provide more food resources and allow *P. vlangalii* individuals to grow faster and surpass the adverse effects of smaller neonate body size, and finally reach a similar or even larger adult body size compared with the low‐elevation site. The estimated growth curve (Figure 4) suggests that, despite a smaller size at birth, high‐elevation juvenile *P. vlangalii* would reach a similar or larger size than low‐elevation juveniles during the third active season. Presumably, parturition occurred naturally in late July and mid‐August at the low‐elevation and high‐elevation sites, respectively (Li et al., 2014); newborns from both populations would become sexual maturity in that season (September and June for the low‐elevation and high‐elevation populations, respectively) according to estimated ages at sexual maturity. Therefore, larger body size of high‐elevation adult *P. vlangalii* is likely to be due to faster growth during the juvenile stages. Similar results have been observed in other lizard species. For example, hatchling *Psammodromus algirus* and *Sceloporus graciosus* from high‐elevation populations is smaller, but grow faster over the active season to reach the same or larger size by the following years compared with those from low‐elevation populations (Iraeta et al., 2006; Sears, 2005).

An individual animal can achieve a relatively large adult size by delaying maturation or prolonging growth period (Angilletta, Niewiarowski et al., 2004; Iraeta et al., 2006). Delayed maturation at a large body size occurs in some other species of reptiles and amphibians (Liao & Lu, 2011; Sears & Angilletta, 2004; Wapstra, Swain, & O'Reilly, 2001). In this study, however, the estimated age at sexual maturity was younger for the high‐elevation population than for the low‐elevation population, which was inconsistent with our third prediction. If delayed maturation occurred in the high‐elevation population, an older age at sexual maturity should be observed. Accordingly, large adult body size for *P. vlangalii* at the high‐elevation site might not be caused by the delayed maturation. Delayed maturation at a larger body size is favored in colder environments where juvenile lizards tend to have higher survivorship (Sears & Angilletta, 2004; Shine & Charnov, 1992; Stearns, 1992). Unfortunately, we were currently unable to determine between‐site difference in juvenile survival rate due to limited mark‐recapture data. However, no obvious difference in juvenile return rate (the proportion of recaptured individuals in total marked lizards) was found between the two study sites (low‐elevation vs. high‐elevation: 22.6% vs. 21.5%), probably implying a similar survival probability in the two populations.

Larger body size can also result from the increased longevity (Morrison et al., 2004; Speakman, 2005). Longevity of lizards from the two study populations (the mean values for estimated age of field‐captured adults both based on the von Bertalanffy growth equation and skeletochronology) suggested that on average, adults were older in the low‐elevation population than in the high‐elevation population. Individuals in a population that live longer should result in greater mean population age (Leclair & Laurin, 2006). Despite having a larger adult body size, high‐elevation individuals did have a shorter longevity than low‐elevation ones, which was inconsistent with our final prediction. Therefore, the longevity is unlikely to be an important factor leading to a larger adult body size for the high‐elevation population. No direct correlation between adult body size and longevity was also found in other reptile and amphibian species (Leclair & Laurin, 2006; Oromi, Sanuy, & Sinsch, 2012; Roitberg & Smirina, 2006). For example, the mountain populations of sand lizards (*Lacerta agilis*) live longer, but have similar adult body size, compared with the lowland populations (Roitberg & Smirina, 2006). Furthermore, environmental oxygen concentration is also considered as a potential factor influencing adult body size of ectothermic animals (Callier & Nijhout, 2014). However, reduced oxygen concentration at the high‐elevation site should produce smaller adult body sizes, which is contrary to our results.

In summary, high‐elevation *P. vlangalii* attained a larger adult body size than low‐elevation ones, which was primarily due to fast individual growth rates that are likely to be induced by local environmental resource. Our results possibly reflected divergent life history strategies between the low‐ and the high‐elevation populations of *P. vlangalii* under different environmental conditions. Higher food availability at the high‐elevation site allowed lizards to be born at a smaller size, grow faster, and attain a similar or even larger size after sexual maturity. Contrarily, larger neonates were produced at the low‐elevation site with less food resources, lived longer, but grew more slowly to a smaller adult size.

## CONFLICT OF INTEREST

None declared.

## AUTHOR CONTRIBUTIONS

WGD conceived and designed the experiments. HLL, CXX, and YTJ performed the experiments. HLL analyzed the data. HLL, JMH, and WGD wrote the manuscript. All authors gave final approval of the manuscript for publication.
